# A comparative analysis of the diagnostic performances of four clinical probability models for acute pulmonary embolism in a sub-Saharan African population: a cross-sectional study

**DOI:** 10.1186/s12890-019-1037-x

**Published:** 2019-12-27

**Authors:** Agnès Esiéné, Joel Noutakdie Tochie, Junette Arlette Mbengono Metogo, Paul Owono Etoundi, Jacqueline Ze Minkande

**Affiliations:** 1 Department of Anesthesiology and Critical Care, Faculty of Medicine and Biomedical Sciences, University of Yaoundé I, Yaoundé, Cameroon; 2 Department of Emergency Medicine, Anesthesiology and Critical Care, Yaounde Central Hospital, Yaoundé, Cameroon; 3 Department of Emergency Medicine, Anesthesiology and Critical Care, Douala General Hospital, Douala, Cameroon; 4 Department of Emergency Medicine, Anesthesiology and Critical Care, Gynaeco-Obstetrics and Paediatric Hospital, Yaoundé, Cameroon

**Keywords:** Pulmonary embolism, Wells score, Simplified wells score, Revised Geneva score, Simplified revised Geneva score, Emergency depatment, Sub-Saharan African

## Abstract

**Background:**

The diagnosis of acute pulmonary embolism (PE) is one of the most challenging in emergency settings where prompt and accurate decisions need to be taken for life-saving purposes. Here, the assessment of the clinical probability of PE is a paramount step in its diagnosis. Although clinical probability models (CPM) for PE are routinely used in emergency departments (EDs) of low-resource settings, few studies have cited their diagnostic performances in sub-Saharan Africa (SSA). We aimed to comparatively assess the accuracy of four CPM in the diagnosis of acute PE in sub-Saharan Africans.

**Methods:**

We carried out a cross-sectional study to compare the sensitivity, specificity, positive and negative predictive values and accuracy of four CPM namely; the Wells, simplified Wells, revised Geneva and the simplified revised Geneva (SRG) Scores to computed tomography pulmonary angiography (CTPA) in all adults patients with suspected PE admitted to the EDs of the Gynaeco-obstetric and Paediatric Hospital of Yaoundé and the Yaoundé Central Hospital in Cameroon between January 1, 2017 and April 30, 2018.

**Results:**

In total, we enrolled 30 patients with clinical suspicion of acute PE. PE was confirmed on CTPA in 16 (53.3%) cases. Their mean age was 53.7 ± 15.5 years and 36.7% were males. All four scores had a diagnostic performance superior to 50% in all criteria assessed. The simplified Wells score had the highest sensitivity (62.5%) followed by the Wells score (56.3%). The SRG score had the highest specificity (71.4%). The score with highest PPV was the SRG score (66.7%) and that with the highest NPV was the Wells score (56.3%). Overall the models with the highest accuracies were the Wells and SRG scores (60% for each).

**Conclusion:**

All CPM had a suboptimal diagnostic performance, perhaps highlighting the need of a more optimal CPM for acute PE in SSA. However, the Wells and the SRG scores appeared to be most accurate than the other two scores in the ED. Hence, both or either of them may be used in first intention to predict PE and guide which ED patients should undergo further investigations in an emergency SSA setting.

## Background

Pulmonary embolism (PE) is the most life threatening complication of venous thromboembolism with a 30-day mortality rate of 14–44% [1–4] and a one-year mortality rate of 21–52% [2, 4, 5]. It poses a significant diagnostic challenge in acute medicine due to the lack of pathognomic symptoms and signs [6]. While fatal PE may be the first presentation of venous thromboembolism [7], the diagnosis of PE could be easily overlooked [8, 9] till a confirming autopsy diagnosis [7]. As a result, physicians have developed a low threshold for clinical suspicion and diagnostic testings [10]. Nonetheless, only 10–15% of patients suspected to have acute PE would be confirmed during further investigations [11]. It is worth to mention that, over-testing results in undue expenditures, and complications such as contrast-induced allergic reactions, contrast-induced nephropathy [12] or radiation-induced solid tumors [13] from multi-detector computed tomography pulmonary angiography (CTPA), currently considered the gold standard diagnostic test for PE [14]. Attempting to remedy the problem of unnecessary testing, several clinical probability models (CPM), among which the most widely used are the Wells [15], simplified Wells [16], Revised Geneva [17] and Simplified Revised Geneva [18] scores, were put forth to guide the choice of diagnostic testing depending on the assessed PE probability stratified as low, moderate or high [14]. Guidelines recommend their use combined with D-dimer measurement to avert patients with a low PE probability from undergoing further investigations, without jeopardizing their safety [14]. This diagnostic approach has been reported to decrease the number of undue CTPA by one-third, with just 1–2% missed patients in the group of low probability [19]. This may be of tremendous economic importance in poor-resource emergency department (EDs) of sub-Saharan Africa (SSA) where multi-detector CTPA is quiet scarce and expensive for the majority of the population [20].

Worldwide, primary healthcare centers and EDs are the first to manage of patients with suspicion of acute PE [21]. Here, a rapid accurate diagnosis of PE is crucial. As aforementioned, diagnosing acute PE commences with probability stratification through CPM to preclude patients with a low PE probability from undue further testings [14, 21]. Although widely externally validated in of European countries and the U.S.A where there were derived [21, 22], the lack of evidence on the diagnostic performances of these CPM from SSA makes their applicability of this region a topic of on-going debates [23]. CPM designed in a particular setting perform less in another geographical area [24–27] due to differences in the prevalence of disease and in physicians’ experience of suspected cases [24]. As such, generalizing the validity of PE’s predictive models to SSA without prior scientific evidence, may be inappropriate given that the black race has a 30–60% increase in the incidence of PE [28–30], as well as a 30% increase in PE-related mortality compared to other ethnicities [31]. Report from northern Africa have demonstrated the Pisa model, Wells score and Revised Geneva Score to be most accurate in clinical prediction of PE [32]. Studies conducted in western countries, show that the Wells score and Revised Geneva Score are most accurate in the prediction of PE in the ED [33]. To the best of our knowledge, only one SSA study carried out in a non-emergency department, a cardiology unit in Burkina Faso showed the Wells and Revised Geneva Scores to have a moderate clinical probability in predicting PE [23]. Hence, we aimed to comparatively evaluate the diagnostic accuracy of the four routinely used CPM in the diagnostic approach of acute PE in SSA. We hypothesized that Wells score could best accurately predict PE in an ED of SSA.

## Methods

The methods were described in a study protocol by the same authors [34].

### Study design and setting

This was a cross-sectional multicenter study carried out in the EDs of the following hospitals: the Yaoundé Gynaeco-obstetric and Paediatric Hospital and the Yaoundé Central Hospital between the 1st January 2017 and 30th April 2018. Both hospital are referral and University Teaching hospitals in the capital city of Cameroon, Yaoundé. The Yaoundé Gynaeco-obstetric and Paediatric Hospital is specialized in the treatment of mother and child diseases, but also critical pathologies of the pregnant mother and child. The unit is managed by a Professor in Emergency Medicine and Anaesthesiology-Critical Care Medicine, three consultant Anaesthesiologists-Intensivists and 16 nurses. The Yaoundé Central Hospital is a referral hospital for all adult male and female diseases. To this end, it has a ED for both medical and surgical critical pathologies. The unit is managed by two Professors in Anaesthesiology-Critical Care Medicine, two consultant Anaesthesiologists-Intensivists, 14 nurses and averagely four resident physicians in Anaesthesiology and Critical Care Medicine. Both EDs have anticoagulants but lack thrombolytic drugs such as streptokinase.

### Patient eligibility criteria

We prospectively enrolled all consenting consecutive patients aged beyond 15 years who presented with clinical suspicion of PE to these two EDs. A case of clinical suspicion was defined as any patient presenting with sudden dyspnoea, chest pain, haemoptysis or syncope. All patients presenting with chest pain and syncope underwent a 12 led electrocardiogram (E.C.G) to rule out or rule in an acute coronary syndrome or heart block at the ED. Those with ECG signs of acute coronary syndrome or heart block were excluded and offered treatment accordingly. We also excluded those who refused to consent, those who did not undergo computed tomography pulmonary angiography to rule in or rule out PE despite clinical suspicion, patients with contraindications to computed tomography pulmonary angiography (haemodynamic instability, dehydration, altered renal function) and all patients with a diagnosis of thromboembolic disease documented prior to admission.

### Sampling method

Assuming a power of 20%, a prevalence rate of PE of 39.7% in Douala, Cameroon [35], the SCHULZ and GRIMES formula was used to obtain a minimum size of our sample of 10 subjects per group. The sampling method was exhaustive and consecutive.

### Definition of clinical probability scores

These fours scores, called clinical prediction scores for PE can be defined as pre-test probability models designed to stratify patients with suspected PE into three distinct groups (low-, intermediate- and high-risk) that correspond to an increasing actual prevalence of CTPA confirmed PE [14]. The Wells score (Table 1) [16] is the most used score in high-income settings where it has been validated extensively using both a three-stratification (low, moderate, or high clinical probability of PE) and a two-stratification (PE likely or unlikely) approach [36]. The Wells score is simple to compute and is based on bedside clinical date that is easy to obtain. On the other hand, the weight of one subjective item (‘alternative diagnosis less likely than PE’) may decrease the inter-observer reproducibility of this score [37, 38]. Both the Wells and the Revised Geneva scores (Table 2) were simplified CPM designed in an attempt to increase their universal adoption and widespread clinical applicability (Table 2) [39, 40]. These simplified versions have equally ben externally validated high-income countries [41, 42].

**Table 1:** The original Wells score and simplified Wells score for pulmonary embolism

Predictive variables	Original Wells score	Simplified Wells score
Previous PE or DVT	1.5	1
Heart rate > 100 bpm	1.5	1
Recent surgery or immobilization	1.5	1
Clinical signs of DVT	3	1
Alternative diagnosis less likely than PE	3	1
Haemoptysis	1	1
Cancer	1	1
	Pretest probability;	Pretest probability;
	0–1: low	≤ 1: PE unlikely (low)
	2–6: moderate	> 1: PE likely (high)
	≥ 7: high	
	Dichotomized score:	
	≤ 4: PE unlikely (low)	
	> 4: PE likely (high)	

*DVT* Deep venous thrombosis, *PE* Pulmonary embolism

**Table 2:** The revised Geneva score and simplified revised Geneva score for pulmonary embolism

Predictive variables	Revised Geneva score	Simplified Revised Geneva score
Age > 65 years	1	1
Active malignancy (or considered cure < 1 year)	2	1
Recent surgery or fracture of the lower limbs within 1 month	2	1
Previous PE or DVT	3	1
Haemoptysis	2	1
Unilateral lower limb pain	3	1
Tenderness on lower limb deep venous palpation and unilateral oedema	4	1
Heart rate		
75–94 bpm	3	1
≥ 95 bpm	5	2
	Pretest probability;	Pretest probability;
	0–3: low	0–1: low
	4–10: moderate	2–4: moderate
	≥ 11: high	≥ 5: high
	Dichotomized score:	Dichotomized score:
	0–5: PE unlikely (low)	0–2: PE unlikely (low)
	≥ 6: PE likely (high)	≥ 3: PE likely (high)

*DVT* Deep venous thrombosis, *PE* Pulmonary embolism

### Study procedure

We approached all consecutive patients admitted for clinical suspicion of PE in order to obtain an informed consent. Using a pilot tested interview administered questionnaire, these were assessed for PE clinically probability using four clinical scores externally validated in high-income countries, before any other test to avoid bias; the original Wells score, the simplified Wells score, the Revised Geneva score and the SRG Score.

Patients were considered to have chronic heart failure, cancer, history of previous DVT or PE, or chronic pulmonary disease if these conditions were known prior to admission. Recent surgery was defined as any surgical procedure performed within the last 4 weeks prior to the patient’s admission. A prolonged journey was defined as one lasting at least 4 hours [43]. Questionnaires were completed and systematically reviewed for completeness before proceeding to further diagnostic testing.

### Diagnostic testing and assessment of potential sources of bias

After assessment of the clinical prediction of PE, all patients with no contraindication for CTPA underwent this test as the reference diagnostic test. The diagnosis of PE was established by CTPA detection of an embolus in the pulmonary blood vessels. Radiologists performing the CTPA were blinded to the results of CPM.

### Data analyses

Using CTPA as the reference diagnostic test, we compared the sensitivity, specificity, positive predictive value, negative predictive value and accuracy of each CPM. Sensitivity was defined by the proportion of patients with CTPA confirmed PE who had a PE likely probability. Specificity was the proportion of patients with CTPA unconfirmed PE who had a PE unlikely probability. The positive predictive value was the proportion of patients with a PE likely score who had CTPA confirmed PE. The negative predictive value was the proportion of patients with PE unlikely probability who had an unconfirmed PE on CTPA. The accuracy of each CPM was the proportion of correct clinical assessments divided by the number of all assessments. Data was entered into EPI Info 3.5.1 statistical software and the threshold of statistical significance was set at 0.05.

## Results

### General characteristics of the study population

**Fig. 1:**
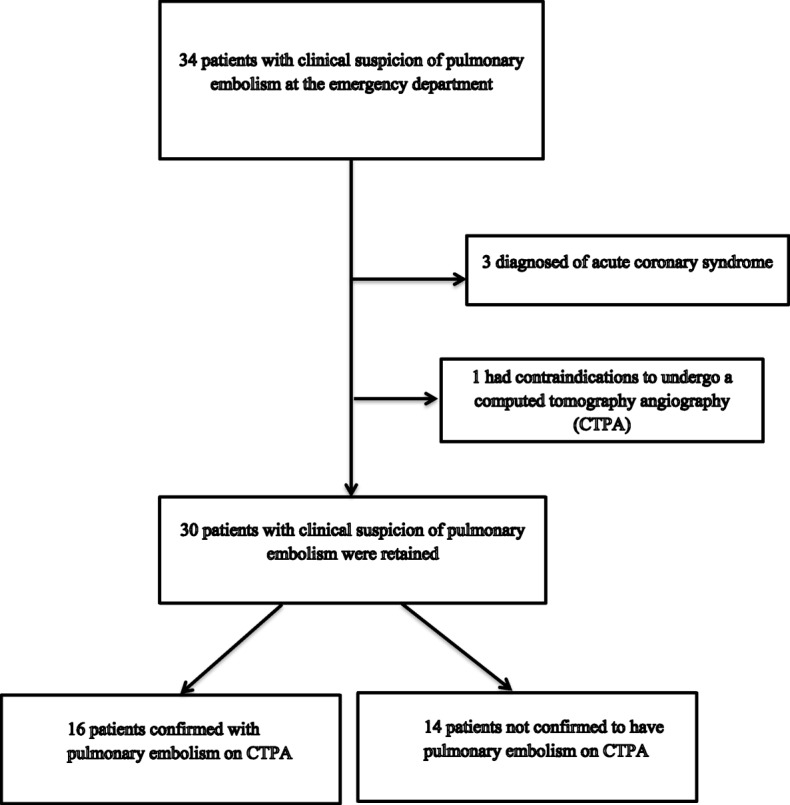
Flow diagram of study selection

We received 34 patients with clinical suspicion of PE. Three patients were excluded due to the diagnosis of acute coronary syndrome and one because he had contraindications (dehydration and a state of shock) for CTPA (Fig. 1). Hence, we enrolled 30 patients with clinical suspicion of PE seen at the ED. PE was confirmed on CTPA in 16 (53.3%) cases. Their mean age was 53.7 ± 15.5 years (range: 32–87 years). About one-third (36.7%) were males, thus, a male to female ratio of 0.57. Dyspnoea was the main reason for ED admission in 86.7% of cases. Patients with confirmed PE and unconfirmed PE were comparable with regards to age, gender, symptoms and signs of PE (Table 3). Obesity was significantly associated with PE. Table 3 summarises the general characteristics of the study population.

**Table 3:** Socio-demographic and clinical characteristics

Groups	Number (%) (*n* = 30)	PE confirmed (*n* = 16)	PE unconfirmed (*n* = 14)	*P* value
Age				
< 65	23 (76.7%)	13	10	0.7431
65–74	4 (13.3%)	1	2	
75–84	3 (10%)	2	2	
Gender				0.3922
Male	11 (36.7%)	7	4	
Female	19 (63.3%)	9	10	
Occupation				0.8177
Employed	23 (76.7%)	12	11	
Unemployed	7 (24.3%)	4	3	
Reason for admission				
Chest pain	11 (36.7%)	7	4	0.3165
Dyspnoea	26 (86.7%)	13	13	0.3524
Haemoptysis	1 (3.3%)	0	1	0.4666
Syncope	3 (10%)	2	1	0.5517
Risk factors				
HIV	5 (16.7%)	3	2	0.5670
Obesity	4 (13.3%)	0	4	0.0365
Prolonged journey	1 (3.3%)	1	0	0.5333
Recent surgery	5 (16.7%)	3	2	0.5670
Active cancer	2 (6.7%)	1	1	0.7241
Past thromboembolism	2(6.7%)	2	0	0.2758
Clinical signs				
Homans sign	9 (30%)	7	2	0.0861
Pulse ≥100	15 (50%)	10	5	0.1361
Hypotension	4 (13.3%)	3	1	0.3677

Table 4 shows the stratification of patients according to the clinical probability of PE and the frequency of PE in three clinical probability categories (low, moderate and high) for each prediction model. The proportions of patients categorized as having low, moderate, or high probability were, respectively: 20, 67, and 13%, for the Wells model; 33, 63, and 4%, for the Revised Geneva model; 40, 53, and 7% for the Simplified Revised Geneva model. The frequencies of confirmed PE in the low, intermediate, and high probability categories were, respectively: 33, 55, and 75% for the Wells model; 50, 53, and 100% for the Revised Geneva model; 50, 62.5, and 0% for the Simplified Revised Geneva model. Table 5 summarizes the diagnostic performance of all four scores.

**Table 4:** Proportion of patients and frequency of pulmonary embolism in the 3 clinical probability categories according to each prediction model

Clinical Probability	Wells score		Revised Geneva score		Simplified Revised Geneva score	
	Patients *N* = 30 (%)	Patients with PE (%)	Patients *N* = 30 (%)	Patients with PE (%)	Patients *N* = 30 (%)	Patients with PE (%)
Low	6 (20%)	2 (33%)	10 (33%)	5 (50%)	12 (40%)	6 (50%)
Moderate	20 (67%)	11(55%)	19 (63%)	10(53%)	16 (53%)	10 (62.5%)
High	4 (13%)	3(75%)	1 (4%)	1 (100%)	2 (7%)	00

**Table 5:** Summary of the diagnostic performances of all four clinical probability models

Models	Sensitivity (%) (95% CI)	Specificity (%) (95% CI)	Positive Predictive Value (%) (95% CI)	Negative Predictive Value (%) (95% CI)	Accuracy (95% CI)
Wells score	56.3 (29.8—80.25)	64.3 (35.14—87.24)	64.3 (44.1—71.6)	56.3 (39.47—71.72)	60 (40.—77.3)
Simplified Wells score	62.5 (35.43—84.8)	50 (23.04—76.96)	58.8 (42.8—73.18)	53.8 (33.91—72.62)	37.43 (37.43—74.54)
Revised Geneva score	50 (24.65—75.35)	64.3 (35.14—87.24)	61.5 (40.45—79.03)	52.9 (37.55—67.79)	56.67 (37.43—74.54)
Simplified Revised Geneva score	50 (24.65—75.35)	71.4 (41.90—91.61)	66.7 (43.31—83.96)	55.5 (40.89—69.31)	60 (40.60—77.34)

## Discussion

This study aimed to determine the diagnostic performances of four bedsides CPM for PE in an emergency setting in SSA. Overall, the models with the highest accuracies were the Well and SRG scores (56.3%).

Two previous reports, the Prospective Investigative Study of Acute Pulmonary Embolism Diagnosis [44] and Prospective Investigation of Pulmonary Embolism Diagnosis [45], emphasized the importance of pre-test for patients with suspected PE. As the aforementioned studies had drawn backs due to standardization, Wells et al. designed a more standardized clinical scoring system in which 66.7, 20.5 and 3.6% of patients with a high, intermediate and low-probability score were diagnosed with PE [16]. Table 6 compares the different clinical probability values obtained with the Wells score from other studies against the present study. Likewise, the Revised Geneva score designed in 2006 showed that 74, 28 and 8% of the ED patients in the high-, intermediate-and the low-probability group had PE [17]. By contrast, using the Revised Geneva score in the present study, we found that 100, 53 and 50% patients with high, intermediate and low-probabilities were confirmed to have PE on CTPA. In a similar study done by Washsh et al. to compare seven CPM (original Geneva score, revised Geneva score, simplified Geneva score, Wells score, simplified Wells score, simplified Charlotte rule, Pisa model) for PE in a chest department in Egypt [32], like in our series, the simplified Wells score equally stood as the scoring system with the highest sensitivity (92% vs. 62.5%). In a study conducted by Kim et al. [46] in the ED, the simplified Wells score had a sensitivity, specificity, PPV and NPV of 4.4, 98.6, 14.3 and 95.0%. By contrast we obtained values of 62.5, 50, 58.8 and 53.8% respectively for the same score. We found a sensitivity, specificity, NPV and PPV for the simplified revised Geneva score of 50, 71.4, 66.7 and 55.5% respectively, contrarily to Kim et al. [46] who obtained 74, 35, 6, and 96%, respectively for sensitivity, specificity, NPV and PPV.

**Table 6:** Comparison of the diagnostic performances of the Wells score

Clinical Probability	At EDs in the present study in Cameroon	Original Wells study [16]	Washsh et al. in Cardiology unit in Egypt [32]	Miniati M et al. in Italy^a^
Low	33%	3.6%	0%	12%
Moderate	55%	20.5%	42.1%	54%
High	75%	66.7%	80%	64%

^a^Miniati M, Bottai M, Monti S. Comparison of 3 clinical models for predicting the probability of pulmonary embolism, Medicine (Baltimore) 2005;84 (2):107–114

From our perspective, apart from being the models with the highest accuracy (60%), the Wells score and SRG score both have the advantage of being purely clinical bedside sores and do not require arterial blood gas sample to be performed compared to more sophisticated scores [17]. The drawback of the Wells score is the “alternate diagnosis less likely than PE” parameter, which adds some degree of subjectivity to an otherwise objective model [47] and, as such, it can hardly be standardized [48]. To overcome this shortcoming of the Wells score, a fully standardized clinical model called the Geneva score, exclusively based on objective parameters was developed [49] and later revised and simplified into the revised Geneva score [17] and the SRG score [40]. Nevertheless, in our series, both the Wells score and the SRG score were found to be the two best scoring systems, with similar accuracy of 60% for ED patients with suspected PE. This finding concurs with that of a recent systematic review which found both model to be comparable in predicting PE [50]. Using the Wells score in our study, the highest proportion of patients (13%) were categorized as having a high clinical probability score and up to 75% of them had a confirmed CTPA PE diagnosis. The frequency of the PE in the low probability category was somewhat higher than that originally reported by Wells [15]. This may be attributed to the higher prevalence of PE in the present study as compared to Wells’ study. Hence, the Wells score is better suited to rule out rather than to rule in the diagnosis of PE, and its performance is likely to be better in a clinical setting where the prevalence of PE is expected to be low [16]. Also, the superiority of the SRG score in our study over the simplified Wells score and the Revised Geneva score may be explained by fully standardized criteria itself and Geneva score-specific variables such as “age > 65 years” and “surgery or fracture within 1 month,” that are absent in the simplified Wells score.

We acknowledge some limitations of our study; firstly its small sample size (*n* = 30), given that simulation studies suggest a minimum of about 100 participants with the outcome of interest (PE) for robust validation studies. However, PE is a rare or often underdiagnosed pathology in Africa, whose prevalence has been recently reported to be low as 0.14% in a systematic review [51]. This may explain the reason for the small sample size in the present study. Secondly, the present study is not a full assessment of prediction models. We basically tested the point-score of four selected prediction models for PE at their recommended thresholds. We kept to this minimal approach, though we fully acknowledge that for full validation of the four models, we need to consider computing the probabilities from which the point-score were derived and validated, rather those probabilities as they are more accurate than the point-score. For instance, measures of discrimination such as c-statistic or area under the curve (AUC) and measures of calibration (calibration plots, Hosmer and Lemeshow statistics, observed/expected event rates, etc.) would have been more accurate. Furthermore, none of the scores portrayed a high diagnostic pre-probability for PE. Hence, it remains questionable whether these four scores are best suitable for the SSA population or is there a need for to develop a scoring system which can predict PE with high accuracy in EDs of SSA. The strengths of this study include: to the best of our knowledge, this is the first study to report the diagnostic performance of four routine bedside CPM for PE in an emergency setting in SSA.

## Conclusion

Grosso modo, all four scores had a moderate diagnostic performance for PE in our setting. Overall, the wells and SRG scores appeared to be more accurate than the other two scores in the ED. Furthermore, the Wells score and the SRGS use only clinical variables, making them easy to use in EDs of Africa. Therefore, both or either scores may be used in first intention in patients seen at the ED with suspected of PE in our resource-limited settings like SSA. These findings may guide clinicians making informed decisions in predicting PE diagnosis and identification of patients at need of further testing or may be anticoagulants therapy in resource-challenged environments where CTPA is not always available or affordable to confirm the diagnosis of PE.

## Data Availability

The datasets used and/or analyzed during the current study are available from the corresponding author on reasonable request”.

## Abbreviations

CPM: Clinical probability model
CTPA: Computed tomography pulmonary angiography
ED: Emergency department
PE: Pulmonary embolism
SRG: Simplified Revised Geneva
SSA: Sub-Sahara Africa
